# The Roles of Arabidopsis C1-2i Subclass of C2H2-type Zinc-Finger Transcription Factors

**DOI:** 10.3390/genes10090653

**Published:** 2019-08-28

**Authors:** Minmin Xie, Jinhao Sun, Daping Gong, Yingzhen Kong

**Affiliations:** 1 Key Laboratory for Tobacco Gene Resources, Tobacco Research Institute, Chinese Academy of Agricultural Sciences, Qingdao 266101, China; 2 Graduate School of Chinese Academy of Agricultural Science, Beijing 100081, China; 3 College of Agronomy, Qingdao Agricultural University, Qingdao 266101, China

**Keywords:** C2H2, C1-2i, ZAT, EAR domain, abiotic stress

## Abstract

The Cys2His2 (C2H2)-type zinc-finger protein (ZFP) family, which includes 176 members in *Arabidopsis thaliana*, is one of the largest families of putative transcription factors in plants. Of the *Arabidopsis* ZFP members, only 33 members are conserved in other eukaryotes, with 143 considered to be plant specific. C2H2-type ZFPs have been extensively studied and have been shown to play important roles in plant development and environmental stress responses by transcriptional regulation. The ethylene-responsive element binding-factor-associated amphiphilic repression (EAR) domain (GCC box) has been found to have a critical role in the tolerance response to abiotic stress. Many of the plant ZFPs containing the EAR domain, such as AZF1/2/3, ZAT7, ZAT10, and ZAT12, have been shown to function as transcriptional repressors. In this review, we mainly focus on the C1-2i subclass of C2H2 ZFPs and summarize the latest research into their roles in various stress responses. The role of C2H2-type ZFPs in response to the abiotic and biotic stress signaling network is not well explained, and amongst them, C1-2i is one of the better-characterized classifications in response to environmental stresses. These studies of the C1-2i subclass ought to furnish the basis for future studies to discover the pathways and receptors concerned in stress defense. Research has implied possible protein-protein interactions between members of C1-2i under various stresses, for which we have proposed a hypothetical model.

## 1. Introduction

### 1.1. Classification of Zinc-Finger Proteins

**Figure 1:**
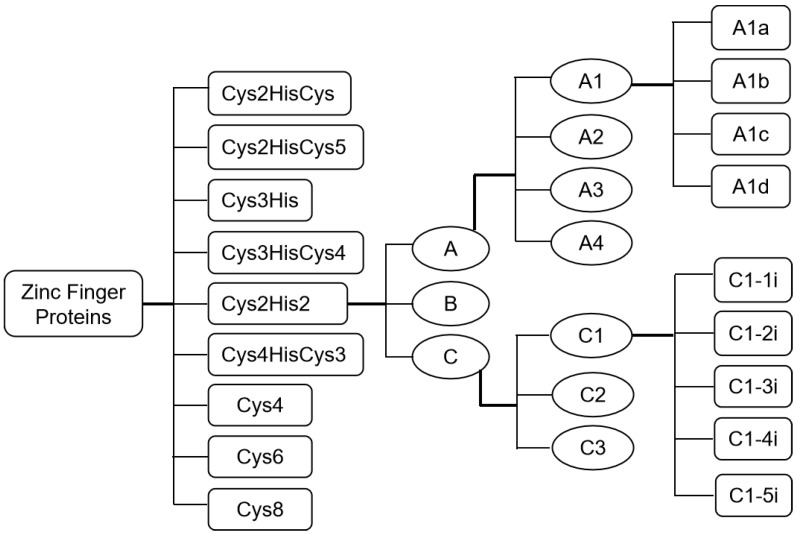
Zinc-finger protein (ZFP) family assignment rules used to identify and assign genes into different subfamilies in *Arabidopsis* [2]. Cys: cysteine; His: histidine; Cys2His2 indicates the presence of two cysteine and two histidine residues in the structure of the ZFP. Cys2His2-type ZFPs are divided into three sets, namely A, B, and C. Set A can be further subdivided into four subsets: A1, A2, A3, and A4. Set C can be further subdivided into three subsets, C1, C2, and C3. The A1 subset is categorized into four subclasses: A1a, A1b, A1c, and A1d, whereas the C1 subset is categorized into five subclasses, namely C1-1i, C1-2i, C1-3i, C1-4i, and C1-5i. 1i indicates one zinc finger, 2i is two zinc fingers, 3i is three zinc fingers, 4i is four zinc fingers, and 5i is five zinc fingers.

Zinc-finger proteins (ZFPs) constitute large protein families, which play important roles in plant development and response to environmental stresses. Based on the order and number of cysteine (Cys) and histidine (His) residues in the structure of the ZFP, as shown in Figure 1, ZFPs can be grouped into nine subfamilies: Cys2His2, Cys2HisCys, Cys2HisCys5, Cys3His, Cys3HisCys4, Cys4HisCys3, Cys4, Cys6, and Cys8 [1,2,3]. Of the nine families, there are 176 members of the C2H2-type ZFP family in *Arabidopsis*, which accounts for a large proportion of all *Arabidopsis* ZFPs. The classical C2H2-type ZFP is composed of an α-helix and an antiparallel β-structure, which contains two Cys and two His residues that hold one zinc ion. All C2H2-type ZFPs of *A. thaliana* are divided into three sets (A, B, and C), with each set being further split into several different subsets, such as C1, C2, and C3 [2,3]. The C1 subset is one of the evolutionarily youngest and largest families, containing 64 members. Many members of subset C2 are involved in the chromatin-remodeling process [2], whereas members of subset C3 are involved in RNA metabolic processes [2]. The C1 subset is related to the processes of development and stress responses. According to the number of dispersed zinc-finger domains, the C1 subset is categorized into five subclasses: C1-1i, consisting of 33 members with one domain in *Arabidopsis*; C1-2i, with 20 members (two domains); C1-3i, consisting of eight members (three domains); C1-4i, with two members (four domains); and C1-5i, consisting of one member (five domains) (Figure 1) [2]. Among these subclasses, the members of the C1-2i subclass have been the most extensively studied and have been shown to be involved in plant development and stress responses; for such research, *Arabidopsis* provides an outstanding model system. In this review, we focus on the roles of the C1-2i subclass in *Arabidopsis* and summarize the research progress that has elucidated the roles of C1-2i members in plant development and response to environmental stresses.

### 1.2. C1-1i Subclass

Over the past few years, many of the 33 members of the C1-1i subclass in *Arabidopsis* have been studied. Previous research has indicated that members, such as glabrous inflorescence stems (GIS, At3g58070; GIS2, At5G06650; GIS3, At1g68360) and zinc-finger proteins (ZFP5, At1g10480; ZFP6, At1g67030; ZFP8, At2g41940), play important roles in controlling trichome initiation and development in *Arabidopsis* [4,5,6,7]. A recent study showed that GIS and GIS2 are regulated by GIS3 [7], and that ZFP5 and ZFP6 act upstream of ZFP8, GIS, and GIS2. Moreover, ZFP8 is a direct target of ZFP5 [5], whereas ZFP5 and ZFP6 mediate trichome initiation by integrating gibberellin (GA) and cytokinin signaling in *Arabidopsis* [6]. ZFP5 also mediates the development of root hairs via the cytokinin and ethylene pathways, and regulates shoot maturation by GA signaling [8]. The trichome-related protein (TRP) negatively regulates trichome initiation-related transcription factors via the GA-signaling pathway, with ZFP5 and ZFP8 being direct target genes of TRP [9]. TCP4 (TEOSINTE BRANCHED 1/CYCLOIDEA/PROLIFERATING CELL FACTOR) is involved in trichome differentiation by positively regulating the *GIS* gene [10].

Other members of the C1-1i subclass have also been investigated. JAGGED (JAG, At1g68480) regulates the development of lateral organs in *Arabidopsis* [11]. The *jag* mutant exhibits narrow petals and abnormal sepals, and JAG is involved in organ growth by controlling cell proliferation [11,12], and by regulating the expression of many genes that are involved in organ growth, such as *CLAVATA 1*, *HANABA TARANU*, *BLADE ON PETIOLE 2*, *KIP-RELATED PROTEIN4* (*KRP4*), and *KRP2* [13]. JAG also acts as an important regulator in the transition from meristem to organ identity [14]. *NUBBIN* (At1g13400), a JAG-like gene, acts redundantly with JAG to regulate the development of lateral organs [15]. RABBIT EARS (RBE) are required in petal development [16], directly repressing TCP4 to control the growth of petal primordia in the early stages of petal development [17]. RBE can also negatively regulate a microRNA164-related pathway that functions in organ-boundary specification [18]. Previous studies had shown that SUPERMAN (SUP, At3g23130) could maintain the boundary between whorls 3 (stamens) and 4 (carpel) of the flower [19]. The latest study presents a new mechanism by which SUP interacts with polycomb group (PcG) proteins, forming a repressor complex to negatively regulate the biosynthesis of auxins at the boundary of whorls 3 and 4 [20]. A recent report showed that the SUP cell autonomously represses the ectopic expression of *APETALA 3* and *PISTILLATA* in whorl 4, and the cell nonautonomously promotes the termination of floral stem cells [21]. Ectopic expression of SUP results in plants with abnormal organs, effects which may be mediated by auxin and cytokinin signaling [22,23]. *KNUCKLES* (*KNU*, At5g14010), a *SUP*-like gene, also promotes the termination of floral stem cells in the floral meristem by repressing the *WUSCHEL* (*WUS*) gene, which is responsible for floral meristem development [24].

Studies have shown that TELOMERASE ACTIVATOR 1 (TAC1, At3g09290), which induces telomerase activity, may play a role in the auxin-signaling pathway [25]. Overexpression of *ZFP2* clearly influences floral organ abscission [26]. ZFP3 acts as a negative regulator involved in regulating light and abscisic acid (ABA) responses in the processes of germination and seedling development [27]. Plants overexpressing ZFP10 (At2g37740) or ZFP11 (At2g42410) result in abnormal phenotypes compared with the wild type (WT), such as dwarfed, aberrant leaf shape and early flowering, the leucine-rich region of these proteins possibly being responsible for the results [28,29]. Transgenic overexpression of the *UPRIGHT ROSETTE* (*URO*, At3g23140) gene can influence homeostasis of the auxin indole-3-3acetic acid (IAA) [30].

### 1.3. C1-3i, C1-4i, and C1-5i Subclasses

The C1-3i subclass includes eight ZFPs, each of which contains three dispersed C2H2-type zinc fingers. Of these, ZAT1 (At1g02030) may be involved in nitrate response [31]. DAZ1 (At2g17180) and DAZ2 (At4g35280) are necessary for sperm cell division and fertility, and the two ethylene-responsive element binding-factor-associated amphiphilic repression (EAR) domains of DAZ1/DAZ2 function as transcriptional repressors in the male germline [32]. The C1-4i subclass contains two members, At1g56200 and At1g49900, both of which have four dispersed zinc fingers [2], of which EMB1303 (At1g56200) is necessary for chloroplast development in *Arabidopsis* [33]. C1-5i subclass contains only one member, with five dispersed zinc-finger domains, but its function is currently unknown [2].

### 1.4. C1-2i Subclass

**Figure 2:**
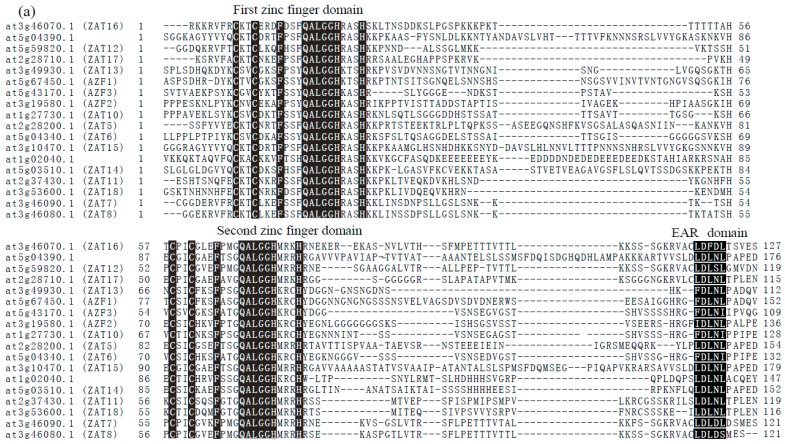

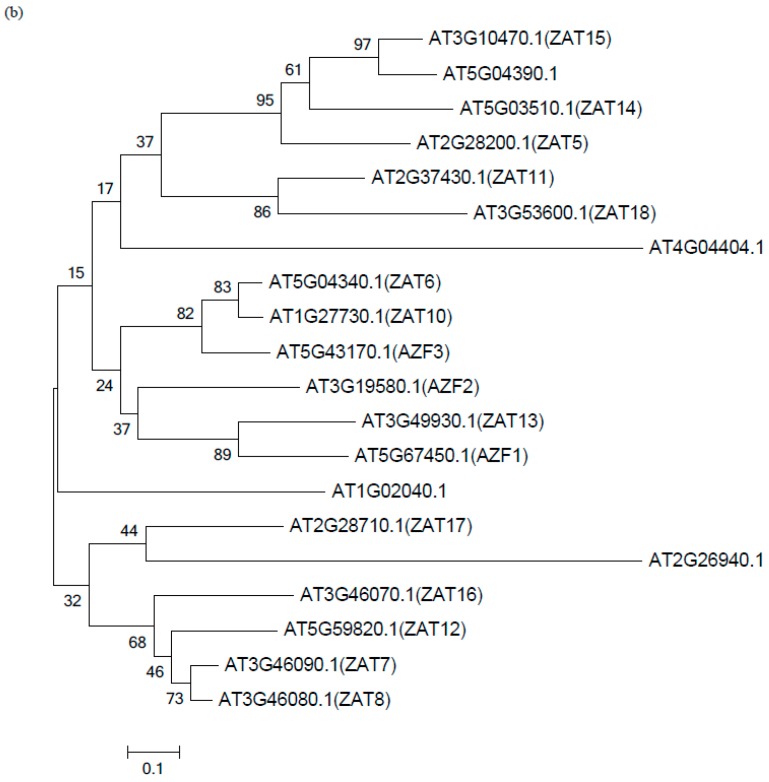
(**a**). Amino acid sequence comparisons of the two zinc-finger regions and the conserved domain in C1-2i subclass of C2H2-type ZFPs in *Arabidopsis*. Sequence alignment was performed with ClustalX2. The first zinc-finger domain, second zinc-finger domain, and the ethylene-responsive element binding-factor-associated amphiphilic repression (EAR) domain were highlighted using a black box; (**b**). Phylogenetic tree of members of the C1-2i subclass of *Arabidopsis* C2H2-type zinc-finger proteins (ZFPs). The phylogenetic tree was constructed using MEGA 5 with neighbor-joining method. The number of bootstrap values was set at 1000 replications. Bootstrap values were provided at each node. The genes of the C1-2i subclass are indicated by their accession numbers, with the corresponding gene names in brackets. AZF: *Arabidopsis* zinc-finger protein; ZAT: zinc finger of *Arabidopsis thaliana.*

The subclass C1-2i contains 20 members, namely AZF1, AZF2, AZF3, ZAT5, ZAT6, ZAT7, ZAT8, ZAT10, ZAT11, ZAT12, ZAT13, ZAT14, ZAT15, ZAT16, ZAT17, ZAT18, At1g02040, At2g26940, At4g04404, and At5g04390. Figure 2b shows the phylogenetic relationships of these members. The 20 ZFPs can be distributed into three main clades, where the genes within each clade are more similar to each other, such as AZF1/2/3, ZAT6, ZAT10, and ZAT13, which may be involved in response to drought, salt, cold, and osmotic stresses, whereas AZF1/2/3 and ZAT10 can recognize the same AGT core sequences of A (G/C) T-X_3~4_-A (G/C) T [34]. Another clade includes ZAT5/11/14/15/18, At4g04404, and At5g04390, whereas the third clade contains ZAT7/8/12/16/17 and At2g26940. Most members of C1-2i subclass are involved in abiotic stress response. There are several conserved regions present in the majority of C2H2 ZFPs identified by multiple sequence alignments. The N-terminal motif contains a short stretch of acidic residues and hydrophobic amino acids before the first finger. The majority of C1-2i members contain highly conserved QALGGH motifs in the α-helical region responsible for DNA binding. Near the N-terminus is located a short motif including a consensus sequence of a B-box (KXKRSKRXR), which is a possible nuclear localization signal. Another motif, with a consensus L-box (EXEXXAXCLXXL), is located after the B-box. The other motif is a short hydrophobic region consisting of core DLNL sequence (DLN-box), the EAR motif, at the C-terminus (Figure 2a) [2,3]. 

The EAR domain, the smallest known repressive domain, was initially found in the APETALA2 (AP2)/ETHYLENE RESPONSE FACTOR (ERF) proteins [35]. ERF proteins can bind to the core sequence of a conserved ethylene-responsive element (GCC box) (AGCCGCC) present in the promoters of some defense-related genes [36]. There are 124 ERF genes in the *Arabidopsis* genome, which are involved in response to cold, pathogens, ethylene, ABA, jasmonic acid (JA), and so on [35,37]. ERF proteins contain two classes. Class I ERFs have been found to act as activators, such as AtERF1, AtERF2, and AtERF5. In contrast, class II ERFs function as repressors of transcription, e.g., AtERF3, AtERF4, AtERF7, and tobacco ERF3 (NtERF3) [35,38]. Many of the plant ZFPs containing the EAR motif have also been shown to function as repressors, such as AZF1/2/3, ZAT7, ZAT10, and ZAT12. This review presents new findings about their roles, as described below.

#### 1.4.1. AZF1/2/3

Sakamoto et al. first cloned the AZF1/2/3 (*Arabidopsis* zinc-finger protein 1/2/3), which all contain two conserved C2H2-type zinc fingers and an EAR motif [39]. Expression analysis showed that all AZFs were mainly expressed in the roots, but AZF1 and AZF2 could be detected at different levels of expression in other organs in *Arabidopsis*. AZF3, in particular, was expressed in the root at a low level, whereas AZF2 was expressed at a high level in flowers [39]. All three AZFs can be induced by ethephon [34]. All AZFs function as transcriptional repressors through the EAR motifs under diverse stress conditions [34,39,40].

All AZFs of *Arabidopsis* are involved in water stress responses [39]. The plant hormone ABA has been shown to play important roles in plant response to water stress and seed germination [41,42,43]. AZF1 was rapidly induced by salt and cold stress but showed only a slight response to ABA treatment; AZF3 did not respond to ABA but was induced by cold stress, whereas AZF2 responded strongly to both salt and ABA treatment. Since the ABA-response element (ABRE) is found in the AZF2 promoter, AZF2 probably responds to water stress through the ABA-dependent pathway [34], so that AZF1 and AZF2 probably regulate the expression of ABA-dependent genes by acting as transcriptional repressors with an EAR domain [34,39]. Kodaira et al. also found that AZF1/2 function as transcriptional repressors, interacting with the promoters of some Small Auxin-Upregulated RNA (SAUR) genes which are early auxin-inducible genes [40]. Attempts to generate transgenic lines overexpressing AZF genes under the control of the CaMV 35S promoter were unsuccessful. This suggests that overexpression of AZF genes may severely suppress the growth of the plants [34].

The first C2H2-type ZFP identified in plants, acting as a DNA-binding protein, was ZPT2-1/EPF1 from petunia (*Petunia* × *hybrida*) [44]. ZPT2-1 and ZPT2-5 were originally identified to bind to the EP2 sequence, which contains two tandemly repeated AGT core sequences separated by 10 bp (AGT-X_10_-AGT) [45]. Studies found that AZF1/2/3 and ZAT10 could recognize the repeated AGT and ACT cores separated by 3 bp [A (G/C) T-X_3~4_-A (G/C) T], and the spacer between the two cores was also important, possibly affecting the binding [34]. The optimum spacer between the binding sites for AZFs and ZAT10 was shorter than that of the petunia ZPT2s. The spacer between the two cores in the target sequence may be an important factor in determining binding affinity, with the long spacer region between the two zinc fingers of the C2H2 peptide possibly being necessary to achieve recognition of the spacer in the target sequence.

#### 1.4.2. ZAT10

ZAT10 (previously named STZ) was first found as a cDNA that rescued the yeast calcineurin-deficient mutant through enhancing its salt tolerance [46]. Similar to the situation with AZF1, ZAT10 was highly expressed in roots and was also expressed in other organs of *Arabidopsis*. Expression of ZAT10 was strongly induced by ABA treatment and various stresses, such as salt, drought, cold, reactive oxygen species (ROS), photo-inhibitory light, and osmotic stresses [34,39,47,48]. As with AZFs, ZAT10 also recognizes the AGT core sequences of A (G/C) T-X_3~4_-A (G/C) T.

Previous studies have proved that ZAT10 functions as a transcriptional repressor because of the presence of an EAR domain [34,35]. Recent studies have shown that ZAT10 plays dual roles as both a positive and a negative regulator in response to environmental stresses. Transgenic plants with constitutively expressed ZAT10 exhibited growth suppression and enhanced adaptation to drought, salt, heat, and osmotic stresses, and the expression of ZAT10 also elevated the transcription of some ROS-responsive genes such as *ASCORBATE PEROXIDASE 1* and *2* (*APX1/2*) and *Fe-SUPEROXIDE DISMUTASE 1* (*FSD1*) [34,47]. Unexpectedly, the *ZAT10* knockout and RNAi transgenic plants appeared to exhibit greater tolerance to salt and osmotic stresses [47].

In *Arabidopsis*, LOS2 is a bifunctional enolase, which can negatively regulate the expression of ZAT10 during cold acclimation [49]. Overexpression of *C-REPEAT-BINDING FACTOR (CBF*) *3* can enhance the expression of *ZAT10* [50], and a decrease in *CBF3* expression in the *ice1* (*INDUCER OF CBF EXPRESSION 1*) mutant led to a decrease in ZAT10 expression under cold-stress conditions [51]. Transient expression analysis suggested that ZAT10 could negatively regulate the expression of *RD29A* (*RESPONSIVE TO DEHYDRATION 29A*), which is a target gene of the CBFs [49]. These results imply that ZAT10 may be a subregulon of CBFs. Moreover, ZAT12 was suggested to negatively regulate the expression of *CBF1–3* genes [52]. Thus, ZAT12 functions upstream of ZAT10 during the cold response.

It is well established that ZAT10 and ZAT12 play important roles in the regulation of antioxidant defense genes, such as *APX1/2* and *FSD1*, which are also involved in salt tolerance [47,53]. Recent evidence shows that molecular hydrogen (H_2_) enhances *Arabidopsis* salt tolerance by regulating the ZAT10/12-mediated antioxidant system and modulating sodium exclusion, and *APX1* and *Salt Overly Sensitive 1* (*SOS1*) are possible target genes of the H_2_ response [54]. These findings suggest that ZAT10 and ZAT12 act in concert with one another in response to different stresses.

A recent study revealed that ZAT10 can directly interact with mitogen-activated protein kinase 3/6 (MPK3/6) in vivo and in vitro, and confirmed that ZAT10 is a direct target substrate for MPK3/6 in *Arabidopsis*, in which two serine residues, located at positions 8 and 210, were verified as phosphorylation sites [55]. A recent report indicated that the phosphorylation activated by MPKs is important for the biological function of ZAT10 in conferring osmotic stress tolerance [55]. The report found that overexpression of *ZAT10* could rescue the defective phenotype of *zat10* in *Arabidopsis*. This finding showed that ZAT10 increased the osmotic stress tolerance function by acting as a positive transcription regulator. Further site-directed mutagenesis experiments proved that the phosphorylation of ZAT10, regulated by MPKs, is indispensable for the osmotic stress response but that the EAR motif is not [56]. Further research is required to identify the regulatory mechanism of ZAT10 in plant defenses.

#### 1.4.3. ZAT12

ZAT12 also has an EAR repression motif and functions as a repressor [22,35]. The full-length cDNA for the *ZAT12* gene responding to light stress was isolated by differential display technique [57]. Recent studies suggest that ZAT12 plays important roles in responses to abiotic stress and ROS. ZAT12 was found to be induced by high light [57], wounding [58], cold [52], heat [59], ROS [59,60], and hydrogen [54]. Overexpressing *ZAT12* in a transgenic plant showed different effects on plant development in three different studies. Iida et al. (2000) reported that overexpression of *ZAT12* enhanced tolerance to high light, but the leaves of the transgenic plants became thicker and dark green [57]. However, Rizhsky et al. (2004) did not find any effect on plant growth in *ZAT12*-overexpressing plants, which exhibited a limited increase in cold tolerance [58], whereas another study found that *ZAT12*-overexpressing lines exhibited increased cold tolerance but showed a defective phenotype, with curled leaves, short petioles, and premature flowering (“bolting”) [52]. This discrepancy in phenotypes of the *ZAT12*-overexpressed plants may reflect differences in *ZAT12* expression in plants between be the three studies. The existence of diverse stress-responsive genes suggests that the response of plants to environmental stresses is very complex.

It is well known that the CBF cold-response pathway, which includes three crucial transcription factors, namely CBF1, CBF2, and CBF3, plays an important role in cold acclimation of *Arabidopsis* [52,61]. The CBF proteins function as transcriptional activators that can recognize the C-repeat (CRT) and dehydration response elements (DRE) [52]. The overexpression of CBF2 and CBF3 can induce many cold-responsive (*COR*) genes [50,52]. Vogel et al. (2005) also reported that ZAT12 was induced in parallel with the CBFs, which regulated the expression of 24 cold-responsive genes, suggesting that ZAT12 might be involved in cold acclimation via a new cold-response pathway. Moreover, ZAT12 was suggested to negatively regulate the expression of *CBF1-3* genes [52].

ZAT12 was reported to be essential for the expression of APX1, ZAT7, and WRKY25 during oxidative stress, all of them being involved in the defense response to oxidative stress. The increased expression of ZAT12, ZAT7, and WRKY25 occurred earlier than that of APX1 during the oxidative stress response [58]. ROS may also be linked with the regulation of iron (Fe) uptake. H_2_O_2_ levels are increased under Fe deficiency, depending on action of the FER-LIKE IRON DEFICIENCY-INDUCED TRANSCRIPTION FACTOR (FIT). FIT plays a key role in Fe uptake through up-regulation of a set of target genes such as *FERRIC REDUCTASE-OXIDASE 2* (*FRO2*) and *IRON-REGULATED TRANSPORTER 1* (*IRT1*) [62]. Studies have found that FIT can directly interact with ETHYLENE-INDUCED 3 (EIN3) and EIN3-LIKE 1 (EIL1), which play positive roles in Fe uptake [63]. ZAT12 was found to be a direct target of EIN3, with overexpression of EIN3 being able to up-regulate the transcript abundance of *ZAT12* [64], and ZAT12 can also interact with FIT, forming a protein complex through its EAR domain, and down-regulating FIT expression as a negative moderator involving H_2_O_2_ [62]. These results suggest that multiple cross-link pathways might be involved in the regulation of FIT. ZAT12 acts as a negative moderator involved in the FIT interaction network, with H_2_O_2_ acting as a signal for ZAT12 and FIT regulation.

#### 1.4.4. ZAT6

A study showed that ZAT6 plays a role in root development and phosphate homeostasis [65], with the expression of *ZAT6* being strongly activated by salt and osmotic stress [66]. Other studies showed that the transcription of *ZAT6* could also be induced by drought, cold, and pathogens. *ZAT6*-overexpressing lines showed increased tolerance to drought, salt, cold, and pathogen stresses, but knockdown plants of ZAT6 exhibited decreased stress tolerance [67]. A recent report found that *ZAT6* expression responds to cadmium (Cd) stress, with plants overexpressing *ZAT6* showing markedly increased tolerance to Cd, whereas the *zat6* mutants exhibited increased Cd sensitivity [68]. Further analysis suggested that ZAT6 positively regulates the expression of *GLUTATHIONE 1* (*GSH1*), *GSH2*, and PHYTOCHELATIN SYNTHASES (*PCS1*) and *PCS2*, which are all involved in Cd accumulation and tolerance. Evidence also revealed that ZAT6 is able to bind to the TACAAT box in the promoter of *GSH1* specifically, and that *GSH2*, *PCS1*, and *PCS2* might not be the direct targets of ZAT6 [68].

In response to cold and pathogen stress, ZAT6 can directly activate the expression of *CBF1–3*, and increase expression of *ENHANCED DISEASE SUSCEPTIBILITY 1* (*EDS1*), *PHYTOALEXIN DEFICIENT 4* (*PAD4*), and *PATHOGENESIS-RELATED GENE 1* (*PR1*), *PR2,* and *PR5* by binding to the TACAAT domain present in their promoters [67]. Further research showed that the melatonin concentration increased significantly in response to cold stress, and melatonin induced the expression of *ZAT6,* which subsequently activated the expression of *CBF*s, with the ZAT6-mediated CBF pathway was required for melatonin-mediated response to cold stress in *Arabidopsis* [69].

Liu et al. (2013) found that ZAT6 is a direct target substrate of MPK6, which can phosphorylate ZAT6 at two sites, namely Ser8 and Ser223, and MPK6-mediated phosphorylation may be essential for ZAT6 in regulating seed germination under salt and osmotic stress [66].

#### 1.4.5. ZAT7

ZAT7 was first identified in response to oxidative stress, and may be involved in *APX1* expression [58]. Transgenic overexpression of *ZAT7* was found to cause growth suppression and to increase the tolerance of plants to salinity stress. The EAR domain of ZAT7 has been shown to be responsible for this increased tolerance to salinity stress, but is not involved in the growth suppression [70]. The findings indicated that the EAR motif of ZAT7 plays an important role in response to abiotic stress in *Arabidopsis*. ZAT7 was reported to interact with WRKY70 and a miRNA transport protein HASTY, with the expression of ZAT7, HASATY, and WRKY70 increasing in *APX1* knockout transgenic plants, which were more tolerant than the WT to salt stress. These results might suggest that the three proteins are involved in a salinity stress-response pathway, and function through forming a complex [70].

#### 1.4.6. ZAT11

Studies have reported that the transcript abundance of *ZAT11* can be highly induced by H_2_O_2_, and *ZAT11* was shown to be involved in paraquat-induced oxidative stress, which leads to programmed cell death [71,72]. GUS activity driven by the promoter of ZAT11 was mainly detected in cotyledons, hypocotyls, and primary roots. *ZAT11*-overexpressing lines produced primary roots that were significantly longer than in the empty vector lines. This suggests that ZAT11 positively modulates the growth of the primary root. Moreover, evidence showed that ZAT11 functions as a negative modulator to repress nickel ion (Ni^2+^) tolerance by directly or indirectly regulating *IREG2*, which is a vacuolar Ni^2+^ transporter gene [73].

#### 1.4.7. ZAT18

Histochemical GUS analysis showed that ZAT18 was strongly expressed in leaves, stems, and siliques, with lower levels of expression in roots and hypocotyls [74]. Subcellular localization analysis suggested that ZA18 was localized in the nucleus. *ZAT18*-overexpressed plants exhibited increased tolerance to drought stress, but the loss-of-function mutant of *ZAT18* exhibited reduced tolerance [74]. RNA sequencing analysis found that 423 and 561 genes had significant changes in *ZAT18*-overexpressed plants before and after drought stress, respectively. The target genes of ZAT18 include some defense genes, such as the senescence/dehydration-associated gene *ERD7*, the cold-related gene *COR47*, the response to ABA and salt 1 gene (*RAS1*), the late-embryogenesis-abundant gene *LEA6*, the jasmonate-domain gene *JAZ7*, and the ABA-receptor gene *PYL5* [74]. The molecular mechanism by which ZAT18 regulates the various target genes in order to respond to complex stresses requires further research.

#### 1.4.8. Other ZFPs of the C1-2i Family

The function of other C2H2-type ZFPs in the C1-2i family is still unknown. The first C2H2-type ZFP reported in a plant was ZPT2-1/EPF1 from petunia (*P. × hybrida*) [44]. The ZAT5 amino acid sequence similarity with ZPT2-1 is 48.2%. The proteins also have similar structures and expression patterns. A study showed that expression of ZAT5 is more organ-specific than ZPT2-1, having the highest amount of open flowers followed by inflorescence stem, flower buds, and siliques [75].

## 2. Conclusions

**Figure 3:**
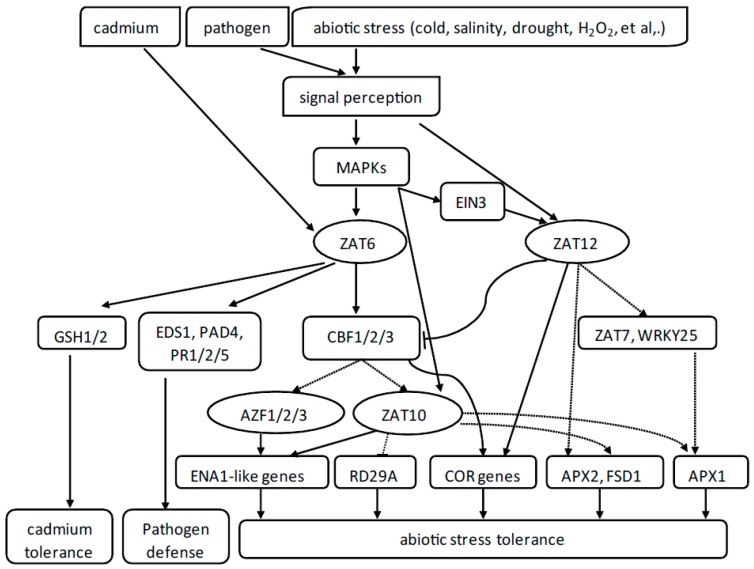
A hypothetical model of links between different members of C1-2i subclass of C2H2-type zinc-finger proteins (ZFPs) from *Arabidopsis*. AZF1/2/3 (*Arabidopsis* zinc-finger protein 1/2/3), ZAT6 (zinc finger of *Arabidopsis thaliana* 6), ZAT7, ZAT10, and ZAT12 are C2H2 zinc-finger transcription factors in the C1-2i subclass. Solid arrows represent positive regulation, T-bars represent negative regulation, and dotted arrows represent indirect regulation. Abbreviations: MAPKs, mitogen-activated protein kinases; CBF, C-repeat binding factor; EIN3, ethylene-induced 3; GSH1/2, glutathione 1/2; EDS1, enhanced disease susceptibility 1; PAD4, phytoalexin deficient 4; PR1/2/5, pathogenesis-related gene 1/2/5; ENA1-like genes, encoding a Na^+^-ATPase-like genes; RD29A, responsive to dehydration 29A; COR genes, cold-responsive genes; APX1/2, ascorbate peroxidase 1/2; and FSD1, Fe-superoxide dismutase 1.

Among the 20 members of the C1-2i subclass, belonging to the C2H2-type ZFPs of *Arabidopsis*, considerable evidence has shown that AZF1/2/3, ZAT6, ZAT7, ZAT10, ZAT11, ZAT12, and ZAT18 play crucial roles in response to various stresses. Most members of the C1-2i subclass, such as AZF1-3, ZAT7, ZAT10, and ZAT12, are considered to function as repressors, using the EAR motif. These studies imply possible protein-protein interactions between different ZFPs under various stresses, for which we have proposed a hypothetical model, shown in Figure 3. Mitogen-activated protein kinases (MAPKs) are serine/threonine kinases that can phosphorylate transcription factors or other kinases. The MAPK signal cascades play key roles in mediating abiotic stress response. There are three classes of kinases which highly conserved in eukaryotes, namely MAPK, MAPK kinase, and MAPK kinase kinase. The MAPKs of *Arabidopsis* can be strongly induced by exposure to stresses such as salt, osmotic, and H_2_O_2_, among others [76,77,78]. The expression of ZAT6 can be strongly induced by salt, drought, cold, pathogen, and osmotic stress [66,67]. ZAT6 can also be phosphorylated by MPK6 at two sites, namely Ser8 and Ser223. ZAT10 has been identified as the direct target substrate of MPK3/6. The activated MPKs regulate the ZAT10 transcription factor by direct phosphorylation, and phosphorylation of ZAT10 might lead to a change in the regulation of the expression of its target genes. EIN3 can also be phosphorylated by MPK3/6 [79], and ZAT12 is a direct target of EIN3 [64], so that ZAT12 may also be involved in MAPK signal cascades. Thus, MAPK-mediated phosphorylation of C2H2-type ZFPs may play important roles in their response to various stresses. As shown in Figure 3, ZAT6 can activate the expression of *CBF1-3* by directly binding to the TACAAT motif in the promoters of *CBF1-3* [67]. ZAT12 was suggested to negatively regulate the expression of *CBF1-3* genes involved in cold acclimation [52]. ZAT12 is also needed for the expression of ZAT7 during oxidative stress [58]. Interestingly, ZAT10 plays dual roles as a positive and a negative regulator in response to environmental stresses, with ZAT10 being a subregulon of *CBF*s [47] and *ZAT12* functioning upstream of *ZAT10* during cold response.

Plants have developed complex mechanisms to alter their responses to various environmental constrains. Transcription factor are important mediators in regulating defensive expression. Although positive regulation mechanisms concerned in plant stress responses are exceedingly well studied, only a few transcription factor function repressors have been characterized, and little is known about what manipulates stress associated gene expression during ordinary growth. It is vital to discover such repression domain for revealing the molecular mechanisms of negative regulation. Recent studies recommend that most members of the C1-2i subclass of C2H2-type ZFPs contain the EAR domain, so as to explore the molecular mechanism of the plant stress defense. Further research ought to be needed to isolate and discover the proteins that interact with EAR domain, so as to explore the molecular mechanism of the repressor with the EAR in the regulation of gene expression. It will assist us to apprehend the function of plant repressors that alters transcription in detail. Moreover the type of target sequence recognition among C2H2-type ZFPs is unique. The length of the spacer between the two zinc fingers of C2H2 peptides can also be an essential component in figuring out binding affinity. It is noteworthy that there are different regions in the C2H2 protein involved in target sequence recognition. These factors have to take into account the highlighting of the target genes. These studies of the C1-2i subclass will grant future understanding about the integral pathways and receptor associated to stress defense. Further research on molecular mechanism of the C2H2-type ZFPs that underlie multiple stress responses is beneficial in grasping the high adaptability of plants during their response to the various environmental conditions.
